# A look back and new beginnings in 2025

**DOI:** 10.2807/1560-7917.ES.2025.30.1.2501091

**Published:** 2025-01-09

**Authors:** Eurosurveillance editorial team

**Affiliations:** 1 European Centre for Disease Prevention and Control (ECDC), Stockholm, Sweden

At the beginning of every year, we provide our readers and contributors with a recount of the past year as well as a look forward to what may come in the year ahead.

In 2024, *Eurosurveillance* featured 143 regular articles, 81 rapid communications, and 21 other items (editorials, letters/responses, meeting reports). Articles were submitted from 73 countries worldwide and we published articles from 40 countries and 5 continents.

Compared with previous years, the number of articles submitted and published on COVID-19/SARS-CoV-2 was expectedly lower, but with 30 published articles COVID-19/SARS-CoV-2 still featured substantially in the journal. Many contributions covered COVID-19 vaccine effectiveness, while others reported on lessons from the pandemic and the impact of measures on the epidemiology of other, mainly respiratory pathogens which resurged after the strict non-pharmaceutical measures against COVID-19 were lifted.

In response to the ongoing mpox outbreak and disease caused by the novel clade Ib strain of the monkeypox virus (MPXV) in several countries in Africa, the World Health Organization declared a public health emergency of international concern in August 2024 [1]. Rapid communications in *Eurosurveillance* reported early on a possible new clade of MPXV [2] and covered the situation in the affected countries, Burundi and the Democratic Republic of the Congo [3-5]. Finally, in November, two short reports described the first two imported cases of clade Ib MPXV in Europe, in Sweden and Germany, and the ensuing public health responses [6,7].

The largest autochthonous outbreak of dengue fever thus far in Europe occurred in Italy. A short report in November provided details on 199 autochthonous cases that occurred in a small coastal city in central Italy between August and early October 2024 [8].

Nirsevimab, a novel long-acting monoclonal antibody, was authorised by the European Medicines Agency (EMA) as prophylaxis for respiratory syncytial virus (RSV) infection in infants in October 2022 [9]. However, only a few countries included it in their vaccination programmes for the 2023/24 northern hemisphere respiratory virus season. Two rapid communications from countries with universal immunisation programmes, Luxembourg [10] and Spain [11], reported an encouraging early uptake and positive impact of the nirsevimab prophylaxis in reducing severe paediatric RSV infections and related hospitalisations at the beginning of 2024.

In addition to the articles published in our journal on important topics such as antimicrobial resistance, healthcare-associated infections, sexually transmitted infections including HIV/AIDS, tuberculosis, food- and waterborne diseases, and different aspects of surveillance of communicable diseases, including avian influenza H5N1, the examples of rapid communications discussed previously demonstrate the strength of *Eurosurveillance* in rapidly providing evidence to support public health action and decisionmaking when outbreaks occur or new public health measures are implemented.

Looking at various metrics, we are pleased that in 2024, *Eurosurveillance* continued to be well placed among the leading journals in infectious diseases and public health. With an Impact Factor of 9.9 for the year 2023 and ranking sixth among 132 journals listed in the Clarivate Journal Citation Reports category, “Infectious Diseases”, *Eurosurveillance* remained the highest ranked open-access journal. The Scopus CiteScore of 32.7 for 2023 puts *Eurosurveillance* at #3/665 in the category “Public Health, Environmental and Occupational Health”, and also among the top 10 in the categories “Infectious Diseases”, “Immunology and Microbiology (miscellaneous)”, “Virology”, “Microbiology”, “Microbiology (medical)” and “Epidemiology”. The SCImago Journal Rank is #86/2,494 in the category “Medicine (miscellaneous)”, #6/134 in “Epidemiology” and #7/656 in “Public Health, Environmental and Occupational Health” (h-index: 131). Google Scholar Metrics places *Eurosurveillance* at #3 in the category “Epidemiology” and #9 in “Communicable Diseases” (h5-index: 102).

When the new European Centre for Disease Prevention and Control (ECDC) Director Dr Pamela Rendi-Wagner took up office in June 2024, she highlighted the need to reinforce and restore trust in science after the COVID-19 pandemic as one of the main challenges ahead for public health. She also called for “*collaborative leadership to invigorate and renew confidence in scientific evidence and independent public institutions*” [12]. A key area for building trust is vaccination. To encourage an exploration of various issues pertaining to vaccination, *Eurosurveillance* announced the annual theme for 2025: Vaccine-preventable diseases in humans – today's challenges and tomorrow's opportunities. We look forward to receiving many interesting and inspiring contributions for the annual theme.

Quality assurance and provision of sound data and evidence are important elements in building trust and enabling taking the right decisions at the right time. The editors of *Eurosurveillance* will continue to work hand in hand with authors during the editing process to provide authoritative and robust scientific data and evidence. However, to enable that process, reviewers and editorial board members as well as experts in our wide network play an important role as partners who help us select the right articles for our audience. We are grateful for their tireless efforts, often provided on short notice and under the simultaneous pressure of their own work. We are aware that providing advice and peer review are not systematically acknowledged in tenure or grant applications. These tasks are undertaken alongside many other obligations, often in an expert’s free time. To value and recognise our peer reviewers’ contributions, we are publishing in this issue a list with the names of the 562 experts who reviewed for *Eurosurveillance* in 2024 [13]. We are aware that many experts have high demands on their time, but we hope to be able to continue having you as supporters and allies in the future.

To further support and thank our peer reviewers, we enlarged our resources and recognition offers. We developed a short manuscript review e-learning series accredited by the Agency for Public Health Education Accreditation (APHEA) together with the ECDC Continuous Professional Development team. They are self-paced and available for free online. Topics include (i) Evaluating completeness of study reporting, (ii) Evaluating diversity in infectious disease epidemiological observational studies, and (iii) Evaluating the study question. Aside from the list of reviewer names, we offer complementary reviewer recognition through the Web of Science reviewer recognition service and continuing medical education (CME) recognition credits for completed reviews through the European Accreditation Council for Continuing Medical Education (EACCME). We also continue to actively encourage co-reviews for early-career researchers as a mentoring and capacity-building activity. Anecdotal feedback from co-reviewers is very positive, and we hope to see an increased uptake this year.

Diversity and inclusion in research and scholarly publishing are important principles to ensure equity and reduce inequalities, which also apply to infectious disease and public health research and reporting. *Eurosurveillance* has several policies and activities aimed at diversity and inclusion, such as: asking authors to consider the use of inclusive language in their submissions, a scheme for involving early-career researchers in the peer review process, removal of access barriers through diamond open-access publishing under a CC BY licence, a key public health message box to explain an article’s important public health findings to a wider audience, and a pan-European editorial board. Moreover, the journal encourages authors to follow the Sex and Gender Equity in Research (SAGER) guidelines [14], and is signatory to the United Nations Sustainable Development Goals Publishers Compact.

In 2024, we audited the male/female composition of the editorial board and our reviewers. While the male-to-female ratio in the editorial board has been well-balanced over the years, the reviewer composition showed that except for 2023, when there was a male-to-female ratio of 1:1, there has been a consistent predominance of male reviewers since 2010; in 2024, the male-to-female ratio was 1:0.8. We will continue to monitor this and hope to achieve a more balanced composition by proactively encouraging female experts to review for *Eurosurveillance*.​ The 2024 *Eurosurveillance* scientific seminar, Diversity and inclusion in research and scholarly publishing featured the presentations “*Towards gender-responsive and inclusive research: leveraging the SAGER guidelines for better science”* and “*Diversity, Equity and Fairness in the Field: translating the Cape Town Statement into practice”.* These were followed by a lively debate. Already at the beginning of 2024, inspired by the *Cape Town Statement on Fostering Research Integrity through Fairness and Equity* [15], the *Eurosurveillance* editorial board endorsed a policy that encourages interdisciplinary collaboration, diversity and inclusion, and supports the principles of fairness and equity, and encourages authors to recognise the important contributions of various actors. We will continue to promote these principles with the help of our board, our colleagues and our wide network of supporters.

To guide our future direction and ensure that *Eurosurveillance* remains relevant and impactful for its audience, we have commissioned an evaluation of the journal and as part of that effort, we are conducting an online survey which is open until mid-January. We thank all those who have already responded to the survey and welcome further input by completing our survey which is open until 20 January 2025.

Public health continuously faces changes and challenges; regardless of the changes and challenges ahead of us, *Eurosurveillance* remains committed to delivering editorially independent, high-quality, inclusive scientific reporting, which is relevant for public health decisionmaking. We appreciate the ongoing funding, logistical support, and encouragement from our publisher, the ECDC and its Director who grants us editorial freedom. And we warmly thank our editorial board and other supporters, including contributors and colleagues, who remain unnamed here, for their continued support and collaboration. Together, we can advance public health by contributing to providing sound data and evidence to assist the prevention and control of communicable diseases for the benefit of the global population.

## References

[1] World Health Organization (WHO). WHO Director-General declares mpox outbreak a public health emergency of international concern. Geneva: WHO; 2024. Available from: https://www.who.int/news/item/14-08-2024-who-director-general-declares-mpox-outbreak-a-public-health-emergency-of-international-concern PMC11376700 39218470

[2] Masirika LM Udahemuka JC Schuele L Ndishimye P Otani S Mbiribindi JB Ongoing mpox outbreak in Kamituga, South Kivu province, associated with monkeypox virus of a novel Clade I sub-lineage, Democratic Republic of the Congo, 2024. Euro Surveill. 2024;29(11):2400106. 10.2807/1560-7917.ES.2024.29.11.2400106 38487886 PMC10941309

[3] Nizigiyimana A Ndikumwenayo F Houben S Manirakiza M van Lettow M Liesenborghs L Epidemiological analysis of confirmed mpox cases, Burundi, 3 July to 9 September 2024. Euro Surveill. 2024;29(42):2400647. 10.2807/1560-7917.ES.2024.29.42.2400647 39421954 PMC11487916

[4] Nzoyikorera N Nduwimana C Schuele L Nieuwenhuijse DF Koopmans M Otani S Monkeypox Clade Ib virus introduction into Burundi: first findings, July to mid-August 2024. Euro Surveill. 2024;29(42):2400666. 10.2807/1560-7917.ES.2024.29.42.2400666 39421956 PMC11487920

[5] Wawina-Bokalanga T Akil-Bandali P Kinganda-Lusamaki E Lokilo E Jansen D Amuri-Aziza A Co-circulation of monkeypox virus subclades Ia and Ib in Kinshasa Province, Democratic Republic of the Congo, July to August 2024. Euro Surveill. 2024;29(38):2400592. 10.2807/1560-7917.ES.2024.29.38.2400592 39301745 PMC11484285

[6] Treutiger C-J Filén F Rehn M Aarum J Jacks A Gisslén M First case of mpox with monkeypox virus clade Ib outside Africa in a returning traveller, Sweden, August 2024: public health measures. Euro Surveill. 2024;29(48):2400740. 10.2807/1560-7917.ES.2024.29.48.2400740 39611206 PMC11605805

[7] de Jong R Schauer J Kossow A Scharkus S Jurke A . Response of the German public health service to the first imported mpox clade Ib case in Germany, October 2024. Euro Surveill. 2024;29(48):2400743. 10.2807/1560-7917.ES.2024.29.28.2400743 39611210 PMC11605798

[8] Sacco C Liverani A Venturi G Gavaudan S Riccardo F Salvoni G Autochthonous dengue outbreak in Marche Region, Central Italy, August to October 2024. Euro Surveill. 2024;29(47):2400713. 10.2807/1560-7917.ES.2024.29.47.2400713 39574388 PMC11583309

[9] European Medicines Agency (EMA). Beyfortus (nirsevimab) An overview of Beyfortus and why it is authorised in the EU. Amsterdam: EMA; 2022. [Accessed 20 Dec 2023]. Available from: https://www.ema.europa.eu/en/documents/overview/beyfortus-epar-medicine-overview_en.pdf

[10] Ernst C Bejko D Gaasch L Hannelas E Kahn I Pierron C Impact of nirsevimab prophylaxis on paediatric respiratory syncytial virus (RSV)-related hospitalisations during the initial 2023/24 season in Luxembourg. Euro Surveill. 2024;29(4):2400033. 10.2807/1560-7917.ES.2024.29.4.2400033 38275017 PMC10986653

[11] López-Lacort M Muñoz-Quiles C Mira-Iglesias A López-Labrador FX Mengual-Chuliá B Fernández-García C Early estimates of nirsevimab immunoprophylaxis effectiveness against hospital admission for respiratory syncytial virus lower respiratory tract infections in infants, Spain, October 2023 to January 2024. Euro Surveill. 2024;29(6):2400046. 10.2807/1560-7917.ES.2024.29.6.2400046 38333937 PMC10853977

[12] Rendi-Wagner P . Protecting public health in Europe: building trust in a challenging world. Euro Surveill. 2024;29(29):2400461. 10.2807/1560-7917.ES.2024.29.29.2400461 39027942 PMC11258948

[13] Eurosurveillance editorial team. Eurosurveillance reviewers in 2024. Euro Surveill. 2025;30(1):2501097. 10.2807/1560-7917.ES.2025.30.1.2501097

[14] Heidari S Babor TF De Castro P Tort S Curno M . Sex and Gender Equity in Research: rationale for the SAGER guidelines and recommended use. Res Integr Peer Rev. 2016;1(1):2. 10.1186/s41073-016-0007-6 29451543 PMC5793986

[15] Horn L, Alba S, Gopalakrishna G, Kleinert S, Kombe F, Lavery JV, et al. The Cape Town Statement on Fostering Research Integrity through Fairness and Equity advocates for fair practice from conception to implementation of research and provides 20 recommendations aimed at all involved stakeholders. 7th World Conference on Research Integrity (7th WCRI); 29 May–1 June 2022, Cape Town, South Africa. Available from: https://www.wcrif.org/guidance/cape-town-statement

